# Association between choroidal structure and retinal fluid location in type 1 neovascular age-related macular degeneration

**DOI:** 10.1038/s41433-026-04825-2

**Published:** 2026-09-21

**Authors:** Valentina Bilbao-Malavé, Miriam de la Puente, Leyre Ochoa-Tejada, Cristina Irigoyen Bañegil, Alexander Anduaga-Beramendi, María Fraga de la Viesca, Sara Llorente-González, Manuel Sáenz de Viteri, María Hernández, Henar Heras-Mulero, Luis Arias-Barquet, Alfredo García-Layana, Sergio Recalde, Jorge González-Zamora

**Affiliations:** 1 Department of Ophthalmology, Hospital Universitario Clínic, Barcelona, Spain; 2 Retinal Pathologies and New Therapies Group, Experimental Ophthalmology Laboratory, Department of Ophthalmology, Universidad de Navarra, Pamplona, Spain; 3 Red Temática de Investigación Cooperativa en Salud: ‘Prevention, Early Detection, and Treatment of the Prevalent Degenerative and Chronic Ocular Pathology’ from (RD24/0007/0015), Ministerio de Ciencia, Innovación y Universidades, Instituto de Salud Carlos III, Madrid, Spain; 4 Department of Ophthalmology, Clínica Universidad de Navarra, Pamplona, Spain; 5 Department of Ophthalmology, Hospital Universitario de Navarra, Pamplona, Spain; 6 Department of Ophthalmology, Hospital Universitario de Bellvitge, Barcelona, Spain; 7 Navarra Institute for Health Research, IdiSNA, Pamplona, Spain

**Keywords:** Macular degeneration, Pathogenesis

## Abstract

**Objectives:**

To correlate choroidal structure parameters with the presence and amount of subretinal fluid (SRF) and intraretinal fluid (IRF) in treatment-naïve neovascular age-related macular degeneration (nAMD) patients with type 1 macular neovascularisation (MNV).

**Methods:**

A retrospective study reviewed clinical records of 96 patients diagnosed with nAMD between 2015 and 2022. Optical coherence tomography (OCT) scans were analysed for choroidal thickness, choroidal vascularity index (CVI), and large choroidal vessel index (LCVI) using ImageJ software with automated binarisation. SRF was manually quantified using embedded OCT tools, and IRF volume was measured using an artificial intelligence-based platform. Patients with type 1 MNV (*n* = 56) were included for fluid-choroidal correlations. Statistical analyses included t-tests, Mann-Whitney U tests, and Spearman correlations (*p* < 0.05).

**Results:**

In type 1 MNV eyes, SRF presence was associated with greater choroidal thickness (*p* = 0.03), and SRF area correlated positively with choroidal thickness (*p* = 0.02) and foveal LCVI (*p* = 0.004). Maximum SRF height correlated with foveal LCVI (*p* = 0.007). IRF presence was linked to reduced choroidal thickness (*p* = 0.0008), and IRF volume inversely correlated with choroidal thickness (*p* = 0.01) and macular LCVI (*p* = 0.002). No significant choroidal changes were observed pre-MNV or compared to fellow eyes.

**Conclusions:**

In treatment-naïve type 1 MNV, choroidal thickness and LCVI exhibit opposite associations with subretinal and intraretinal fluid, emphasising distinct pathophysiological mechanisms: choroidal venous overload may be associated with SRF accumulation, while choroidal thinning may be implicated in IRF formation. These findings may help generate hypothesis regarding variability in anti-VEGF treatment response and suggest that choroidal characteristics warrant further investigation as potential biomarkers for future individualised management strategies.

## Introduction

Neovascular age-related macular degeneration (nAMD), although less prevalent than the atrophic form of the disease, remains the leading cause of severe vision loss in patients with AMD [1]. Currently, the presence of subretinal fluid (SRF) or intraretinal fluid (IRF) in eyes with nAMD serves as the main indicator of angiogenic activity, guiding the use of anti-angiogenic therapies [2]. However, despite the equivalence attributed to both types of fluid during therapeutic decisions, in recent years, it has been demonstrated that fluid location is associated with different visual prognosis and anatomical outcomes [3]. Specifically, IRF is associated with poorer visual outcomes and a higher prevalence of fibrosis and atrophy, reflecting its association with more aggressive disease progression and structural damage. In contrast, SRF is often better tolerated and maintains stable vision [4].

Despite these findings, the pathophysiological mechanisms underlying SRF and IRF accumulation in nAMD remain poorly understood. Recent studies on other retinal pathologies have highlighted choroidal structural characteristics, suggesting that venous overload and choroidal vascular remodelling may contribute to SRF accumulation [5]. We hypothesise that a similar mechanism may apply in nAMD. This could explain the distinct clinical profiles of SRF and IRF, the persistence of fluid in nearly half of patients treated with anti-angiogenic therapies [6–9], and the association of type 1 macular neovascularisation (MNV) with persistent SRF, which typically requires more intensive anti-angiogenic treatment compared to other MNV types [10].

The aim of this study is to correlate choroidal structure parameters with the presence of SRF and IRF in treatment-naïve nAMD patients with type 1 MNV. Elucidating the complex interplay between choroidal characteristics and fluid location in nAMD could enhance our understanding of treatment response and prognosis, ultimately providing a basis for future studies exploring individualised therapeutic approaches for these patients.

## Materials and methods

A retrospective observational study was conducted. Clinical records of patients diagnosed with treatment-naïve nAMD at two tertiary care hospitals in Spain (Clínica Universidad de Navarra, Pamplona, and Hospital Universitario de Bellvitge, Barcelona) between January 2015 and January 2022 were reviewed. The study was approved by the Ethics Committee of Navarra (EO_2024/10) and adhered to the principles of the Declaration of Helsinki.

Patients aged over 65 years with MNV secondary to AMD confirmed by optical coherence tomography angiography (OCT-A) were enrolled. This age threshold was selected to minimize the inclusion of pachychoroid neovasculopathy, which predominantly affects younger patients (mean age ~60 years). Furthermore, restricting inclusion to patients ≥65 years allowed us to focus on the senescence-associated pathophysiology of AMD. We included only those eyes that exhibited intermediate dry AMD at initial presentation and converted to neovascular AMD during the follow-up. In patients with bilateral type 1 MNV, only one eye was randomly selected for inclusion to ensure independence of observations and avoid bias from intraclass correlation between fellow eyes. Exclusion criteria included advanced AMD with geographic atrophy or fibrosis; concomitant systemic or ocular conditions (e.g., diabetes mellitus, glaucoma, high myopia, fovea plana, macular holes, epiretinal macular membranes, macular telangiectasias, retinal vascular thrombosis, vitreoretinal traction, and other macular diseases); ocular surgery within the preceding year; or prior intravitreal or topical treatments. To exclude patients with pachychoroid spectrum disorders, which can also co-occur with MNV, individuals with choroidal thickness over 300 µm were excluded. Beyond the thickness threshold, all OCT scans were qualitatively reviewed by two experienced retina specialists to identify overt morphological features of pachychoroid-spectrum disease, and eyes exhibiting such features were excluded from the analysis. Additionally, OCT scans with acquisition or segmentation errors due to macular haemorrhages, choroidal clefts, media opacities (e.g., cataracts or vitreous haemorrhages), or poor image quality were excluded from analysis.

**Fig. 1:**
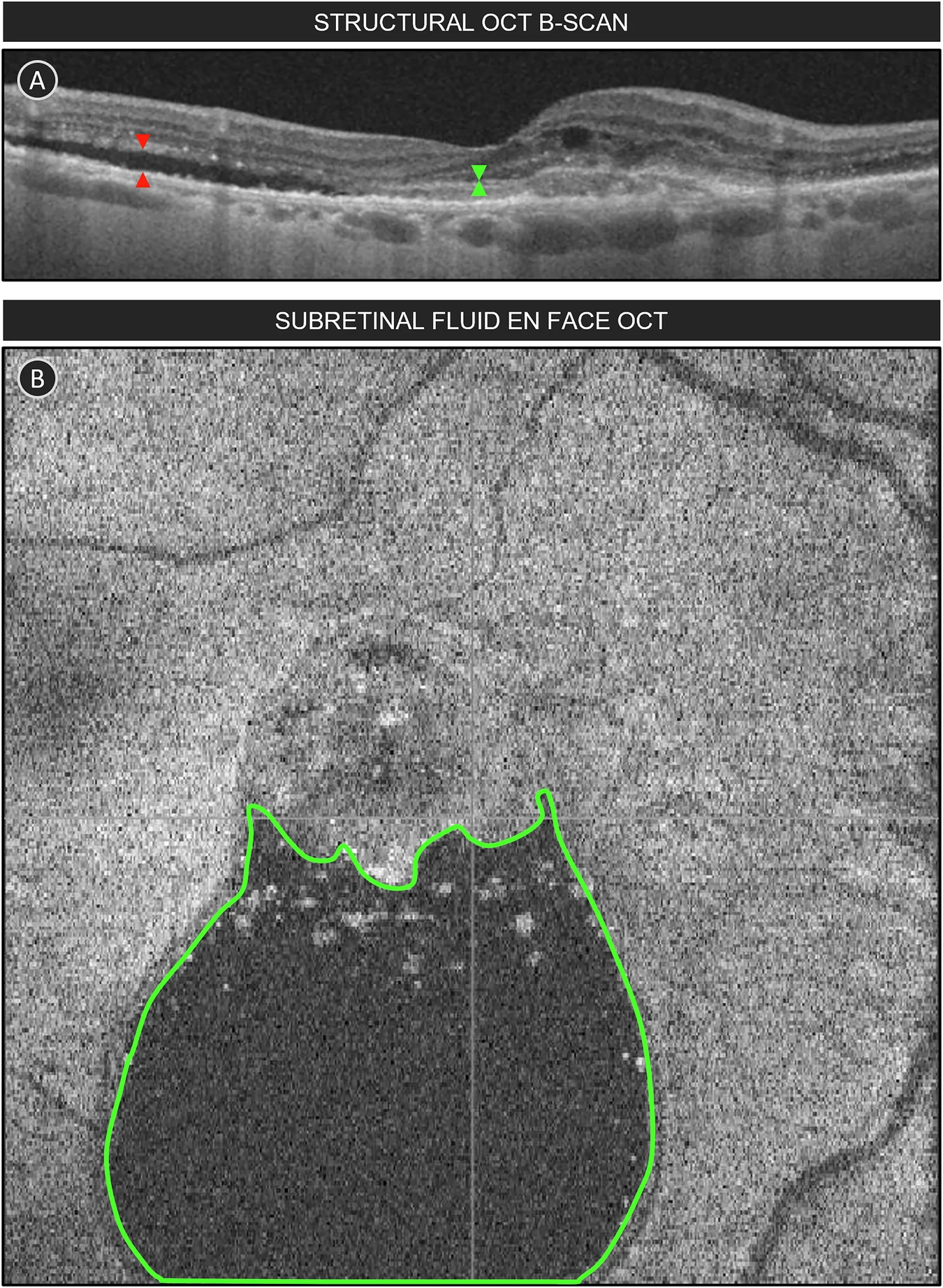
Quantitative measurements of subretinal fluid (SRF) using structural and en face optical coherence tomography (OCT). **A** Macular B-scan showing subfoveal SRF height (green arrowheads; absent in this case) and maximum SRF height, located temporal to the fovea (red arrowheads). **B** En face OCT reconstruction at the retinal pigment epithelium (RPE)/outer segments (OS) level, with the SRF area outlined in green.

Best-corrected visual acuity (BCVA) and anatomical data from OCT scans of both eyes were recorded at the time of nAMD diagnosis. Macular imaging was performed using the DRI OCT Triton swept-source OCT (SS-OCT) Angio system (Topcon Medical Systems, Inc., Oakland, NJ, USA). Structural OCT scans (6 ×6 mm) were analysed, and central retinal and choroidal thickness (within the central subfield of the Early Treatment Diabetic Retinopathy Study [ETDRS] grid) were measured using the system’s automated segmentation and measurement software. SRF was manually quantified by two independent, experienced operators using the embedded OCT calliper tool to measure subfoveal SRF height, maximum SRF height within the macular cube (Fig. 1A), and SRF area in en face reconstructions at the level of the retinal pigment epithelium (RPE)/outer segments (OS). (Fig. 1B). Interobserver agreement for the manually acquired SRF measurements was excellent for all parameters: subfoveal SRF height (ICC = 0.991; 95% CI, 0.982–0.995), maximum SRF height (ICC = 0.975; 95% CI, 0.951–0.987), and SRF area (ICC = 0.993; 95% CI, 0.987–0.997). The mean value of both operators was used for all subsequent analyses. IRF was quantified using an artificial intelligence-based platform (Discovery OCT Fluid and Biomarker Detector, RetinAI AG, Switzerland), which provided automated detection and volumetric measurements.

**Fig. 2:**
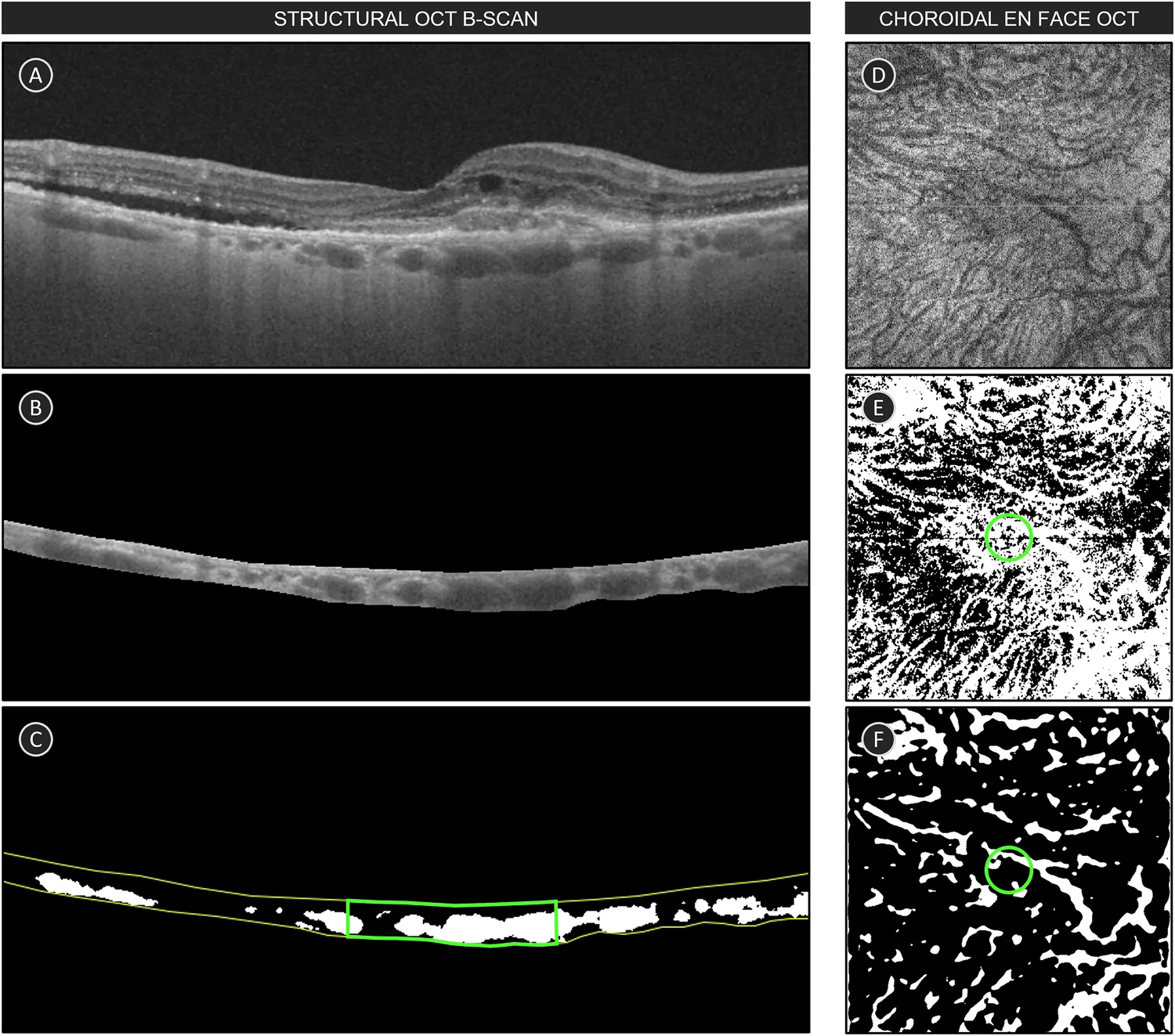
Quantitative measurement of choroidal parameters using structural and en face optical coherence tomography (OCT) with ImageJ software. **A** Macular B-scan OCT. **B** Manual segmentation of the choroidal area. **C** Automated binarisation highlighting choroidal vessel lumens in white; the green rectangle denotes the 1-mm foveal area. **D** En face macular reconstruction at mid-depth choroidal thickness. **E** Binarised en face image showing all choroidal vessel lumens in white, used to calculate the choroidal vascularity index (CVI); the green circle denotes the foveal area. **F** Binarised en face image showing large choroidal vessel lumens in white, used to calculate the large choroidal vessel index (LCVI); the green circle denotes the foveal area.

In addition to central choroidal thickness, the choroidal vascularity index (CVI, defined as the percentage of choroidal luminal area relative to total choroidal area) and the large choroidal vessel index (LCVI, defined as the percentage of large choroidal vessel area relative to total choroidal area) were measured. All choroidal parameters were analysed within two predefined regions: the foveal region, defined as the central 1-mm diameter area, and the macular region, defined as the entire choroidal area within the 6 ×6 mm OCT acquisition cube. CVI was calculated from two complementary sources: the horizontal foveal B-scan and the en face macular reconstruction. For the en face reconstruction, a 10-µm-thick slab centred at the mid-depth of the choroidal thickness was used. For the foveal B-scan, CVI was determined using a previously validated algorithm [11] in ImageJ software (National Institutes of Health, Bethesda, MD, USA): the choroidal area was manually delineated, followed by automated binarisation to identify choroidal vessel lumens (displayed in white), and the lumen-to-stroma ratio was calculated (Fig. 2A–C). For the en face reconstruction, CVI was measured using a custom ImageJ algorithm developed by our team: a smoothing filter was applied to the grayscale en face image, followed by binarisation using a constant threshold to identify all choroidal vessel lumens. For LCVI, the same smoothing filter was applied to the grayscale en face image, followed by binarisation using the auto local threshold method ‘Phansalkar,’ which selectively identifies large choroidal vessel lumens. Both CVI and LCVI were measured within the foveal region (central 1-mm area) and across the macular choroidal area (6 ×6 mm). (Fig. 2D–F).

A total of 96 patients were enrolled. They were further categorised by MNV type (type 1, type 2, or type 3) based on OCT-A findings, as determined by two independent retina specialists. In cases of disagreement, a third retina specialist reviewed the scans for final classification. For the analysis of fluid and choroidal structure, patients with type 1 MNV were included, yielding a total of 56 patients. Choroidal parameters were also compared between the affected eye and the fellow eye in cases without contralateral MNV. Choroidal thickness changes between the OCT scan obtained before the development of MNV and the OCT scan acquired on the day of nAMD diagnosis were assessed.

The Kolmogorov-Smirnov test was used to assess the normality of variable distributions. For normally distributed quantitative variables, comparisons were conducted using the independent Student’s *t* test. For non-normally distributed variables, the Mann-Whitney U test was applied. For paired nonparametric quantitative variables, the Wilcoxon signed-rank test was employed. Fisher’s exact test was used to compare categorical data between groups. One-way analysis of variance (ANOVA) was utilised for multiple comparisons. For correlations between variables, Pearson’s correlation coefficient was used for parametric distributions, while Spearman’s correlation coefficient was used for nonparametric distributions. Interobserver agreement for manual measurements was evaluated using the intraclass correlation coefficient (ICC), demonstrating good-to-excellent reliability; the mean value of both operators was used for all subsequent analyses. Pairwise effect sizes between fluid subgroups were calculated using Cohen’s d, with values of 0.2, 0.5, and 0.8 interpreted as small, medium, and large effects, respectively.

To assess whether the association between IRF and choroidal thickness was independent of potential confounders, a multiple linear regression model was constructed with subfoveal choroidal thickness as the dependent variable and IRF status, age, sex, and baseline BCVA as independent predictors. Model assumptions were verified by assessing normality of residuals (Shapiro-Wilk test), homoscedasticity (Breusch-Pagan test), and multicollinearity (variance inflation factor, VIF). Additionally, an analysis of covariance (ANCOVA) was performed to compare adjusted choroidal thickness means between IRF and non-IRF eyes after controlling for age, sex, and BCVA. A binary logistic regression model was used to identify independent predictors of IRF presence.

Statistical significance was set at a *p*-value < 0.05 (two-tailed). All analyses were performed using GraphPad Prism software version 5.0 (GraphPad Software Inc., San Diego, CA).

## Results

Demographic and baseline characteristics of the 96 patients with treatment-naïve nAMD, categorised by MNV type, are presented in Table 1. Significant differences were observed for age (*p* = 0.037), with older patients in type 2 and type 3 MNV compared to type 1. Retinal thickness also varied significantly (*p* = 0.007), being greater in type 2 and type 3 MNV compared to type 1. Additionally, the presence of IRF showed significant differences (*p* < 0.001), with a higher prevalence in type 3 and type 2 MNV compared to type 1. No significant differences were found for gender, hypertension, BCVA, foveal choroidal thickness, or presence of SRF.

**Table 1:** Demographic and baseline characteristics of patients with treatment-naïve neovascular age-related macular degeneration (nAMD) by macular neovascularisation (MNV) type.

	Type 1 MNV	Type 2 MNV	Type 3 MNV	*p*-value	Total sample
Sample size, *n*	56	29	11		96
Age (years) (mean ± SD)	77.67 ± 8.16	81.94 ± 7.42	82.55 ± 9.96	**0.037**	79.52 ± 8.37
Gender female, *n* (%)	23 (48.28%)	14 (51.72%)	8 (72.72%)	0.154	45 (46.88%)
Arterial hypertension, *n* %	25 (44.64%)	16 (55.17%)	8 (72.73%)	0.203	49 (51.04%)
BVCA (mean ± SD)	0.49 ± 0.27	0.36 ± 0.26	0.34 ± 0.17	0.054	0.51 ± 0.27
Retinal thickness (μm) (mean ± SD)	272.53 ± 69.38	342.73 ± 128.10	343.45 ± 117.17	**0.007**	304.3 ± 103.04
FCT(μm) (mean ± SD)	157 ± 69.87	137.76 ± 73.91	148.27 ± 85.46	0.514	150.19 ± 72.66
Presence of IRF, *n* (%)	19 (33.93%)	21 (72.41%)	11 (100%)	**<0.001**	51 (53.13%)
Presence of SRF, *n* (%)	48 (85.71%)	23 (79.31%)	7 (63.63%)	0.218	78 (81.25%)

Statistically significant *p*-values are reported in bold. *P*-values were calculated using one-way analysis of variance (ANOVA) for continuous variables and Fisher’s exact test for categorical variables.

*BCVA* best corrected visual acuity, *MNV* macular neovascularisation, *FCT* foveal choroidal thickness, *IRF* intraretinal fluid, *SRF* subretinal fluid, *SD* standard deviation.

**Fig. 3:**
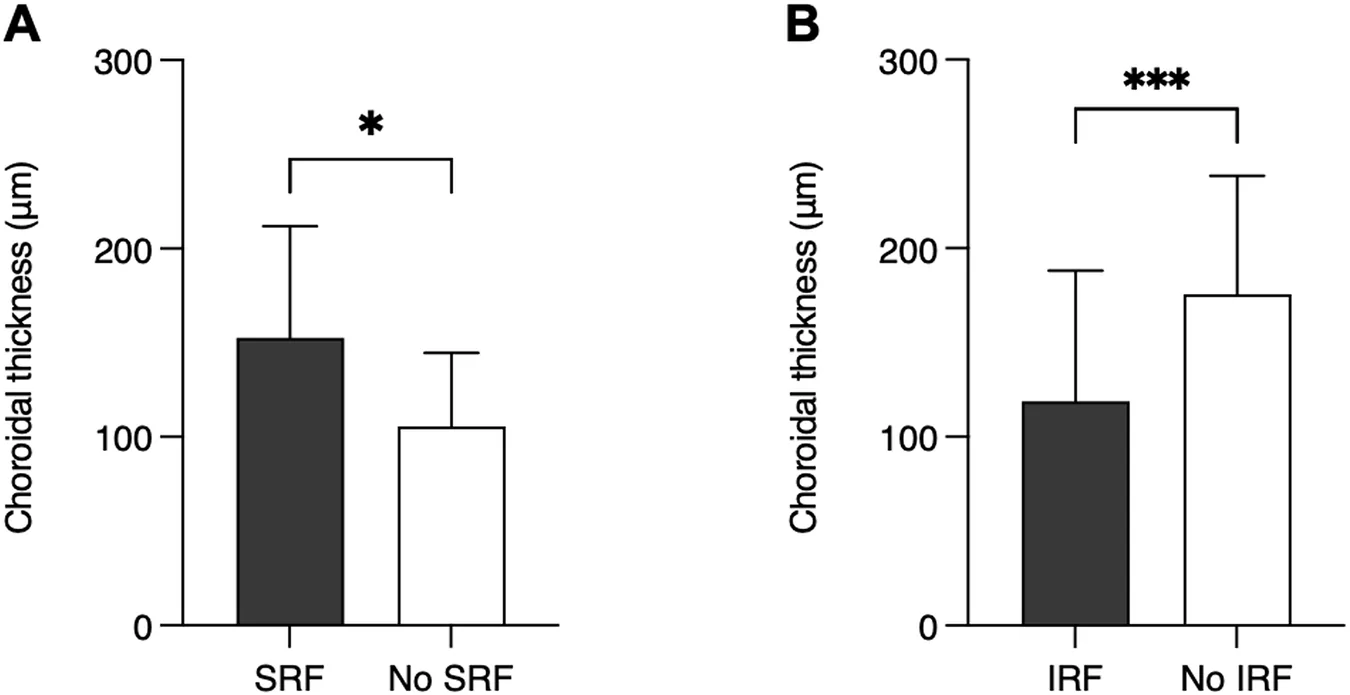
Relationship between choroidal thickness (µm) and retinal fluid location in patients with type 1 macular neovascularisation (MNV). **A** Comparison of choroidal thickness in eyes with subretinal fluid (SRF) and without subretinal fluid (No SRF), showing a significant increase in choroidal thickness in eyes with SRF. **B** Comparison of choroidal thickness in eyes with intraretinal fluid (IRF) and without intraretinal fluid (No IRF), indicating a highly significant decrease in choroidal thickness in eyes with IRF. The error bars represent standard deviation. *p < 0.05, ***p < 0.001.

**Fig. 4:**
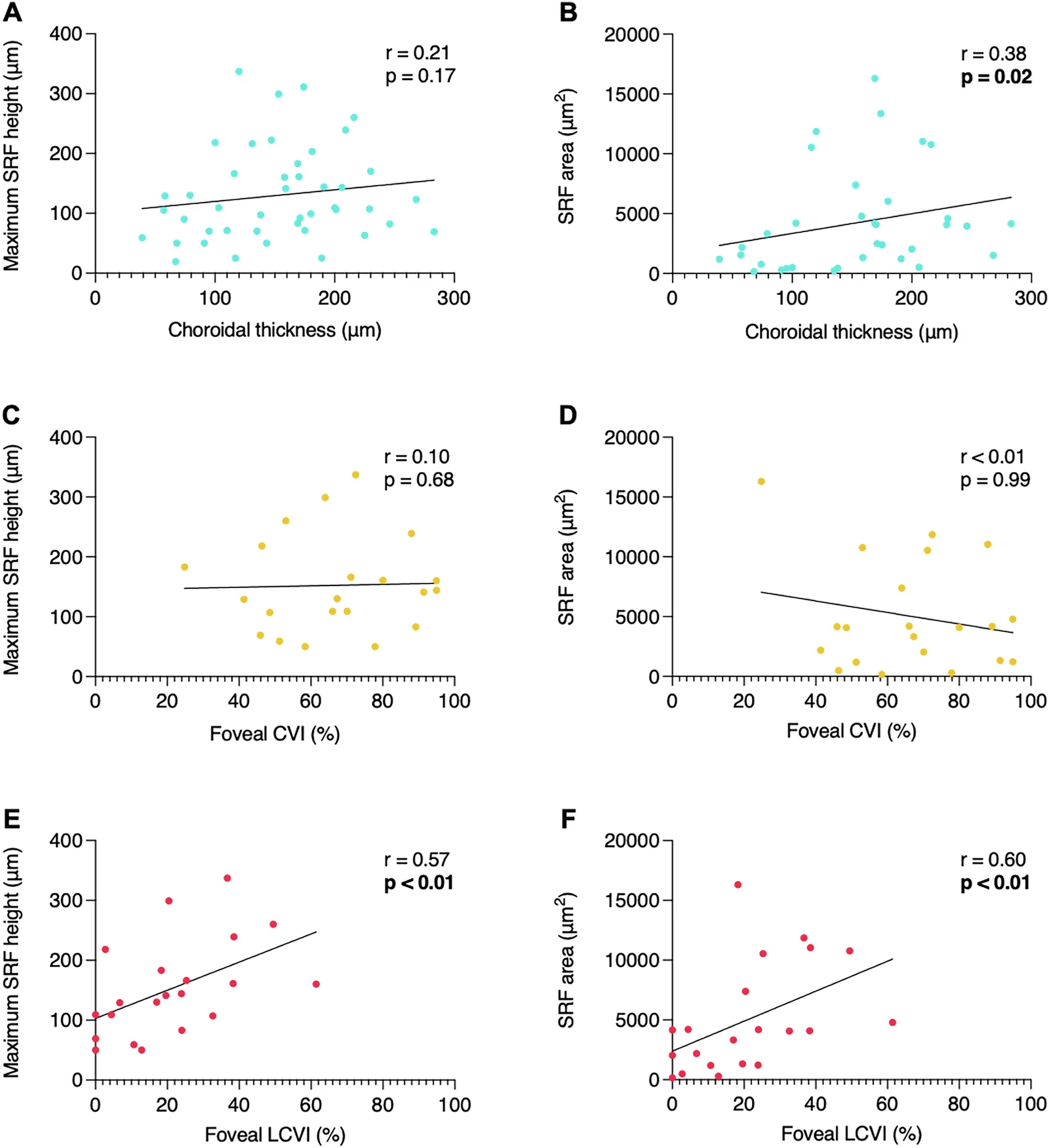
Correlations between choroidal structure parameters and subretinal fluid (SRF) in patients with type 1 macular neovascularisation (MNV). **A** Correlation between choroidal thickness (µm) and maximum SRF height (µm), showing a weak positive association. **B** Correlation between choroidal thickness and SRF area (µm^2^), indicating a statistically significant positive association. **C** Correlation between maximum SRF height and foveal choroidal vascular index (CVI), showing no significant association. **D** Correlation between SRF area and foveal CVI, indicating no significant association. **E** Correlation between maximum SRF height and foveal large choroidal vessel index (LCVI), showing a statistically significant positive association. **F** Correlation between SRF area and foveal LCVI, indicating a statistically significant positive association. Statistically significant *p*-values are reported in bold. *R*-values represent Spearman’s rank correlation coefficient.

The mean subfoveal choroidal thickness in Type 1 MNV patients with SRF was 152.6 μm, compared to 105.7 μm in those without SRF. The difference between these two groups was statistically significant (*p* = 0.03), indicating greater choroidal thickness in the SRF subgroup (Fig. 3A). A positive correlation was observed between choroidal thickness and SRF area (*p* = 0.02) (Fig. 4B), but not with the maximum SRF height (Fig. 4A) nor with the subfoveal SRF height. Additionally, a greater foveal LCVI correlated with greater maximum SRF height (*p* = 0.007) (Fig. 4E) and with the SRF area (*p* = 0.004) (Fig. 4F). However, subfoveal SRF height, maximum SRF height, and SRF area showed no significant correlation with either CVI (Fig. 4C, D) or macular LCVI.

In contrast, IRF was associated with reduced choroidal thickness (*p* = 0.0008) (Fig. 3B). The IRF volume demonstrated an inverse correlation with choroidal thickness (*p* = 0.01) and with macular LCVI (*p* = 0.002). The CVI and the foveal LCVI showed no significant correlation with IRF. Patients with IRF were significantly older than those without IRF (mean age 83.5 ± 7.4 vs 75.1 ± 8.6 years; *p* = 0.005). To evaluate whether the observed association between IRF and choroidal thinning could be attributed to age-related confounding, multivariate analyses were performed. In a multiple linear regression model with choroidal thickness as the dependent variable, IRF status was the only statistically significant independent predictor (*β* = −67.7 μm; 95% CI: −111.1 to −24.4; *p* = 0.003), while age was not independently associated with choroidal thickness after adjusting for IRF (*β* = −1.1; *p* = 0.33). The model explained 39% of the variance in choroidal thickness (R² = 0.39), and all assumptions were met (Shapiro-Wilk *p* = 0.28; Breusch-Pagan *p* = 0.32; all VIF < 10). ANCOVA confirmed that the effect of IRF on choroidal thickness persisted after adjustment for age, sex, and BCVA (*F* = 10.1; *p* = 0.003), with adjusted means of 103.6 μm for IRF eyes vs 171.3 μm for non-IRF eyes. In a logistic regression model for IRF presence, age was a significant independent predictor (OR = 1.14 per year; 95% CI: 1.02–1.28; *p* = 0.025), whereas baseline BCVA and sex were not, suggesting that while older age is associated with a higher likelihood of IRF, the choroidal thinning observed in IRF eyes reflects a distinct structural phenotype not attributable to age alone.

Among the 56 eyes with type 1 MNV, the distribution of fluid subtypes was as follows: 37 eyes (66.1%) exhibited isolated SRF, 8 eyes (14.3%) exhibited isolated IRF, and 11 eyes (19.6%) exhibited both SRF and IRF. Choroidal parameters differed across these subgroups. Eyes with only SRF showed the greatest mean subfoveal choroidal thickness (169.6 ± 56.9 μm) and the highest LCVI (25.6 ± 17.7%). In contrast, eyes with only IRF and eyes with both fluid types presented similarly reduced choroidal thickness (105.7 ± 38.9 μm and 103.7 ± 52.5 μm, respectively) and LCVI (7.4 ± 6.4% and 9.6 ± 7.7%, respectively). Pairwise effect sizes confirmed large differences between SRF-only and IRF-only eyes for both subfoveal choroidal thickness (Cohen’s d = 1.18) and LCVI (*d* = 1.09), as well as between SRF-only and both-fluid eyes for subfoveal choroidal thickness (*d* = 1.18) and LCVI (*d* = 1.06). Subfoveal choroidal thickness differences between IRF-only and both-fluid eyes were negligible (*d* = 0.04), while LCVI showed a small effect size (*d* = 0.39), suggesting that the presence of IRF, with or without concomitant SRF, is associated with a similarly reduced choroidal phenotype. CVI did not follow a consistent pattern across subgroups (SRF only: 40.1 ± 8.0%; IRF only: 43.6 ± 3.7%; both: 35.0 ± 8.3%), consistent with the absence of significant correlations between CVI and fluid parameters observed in the main analysis.

There was no significant difference in choroidal thickness between the OCT scan obtained before the development of MNV and the OCT scan acquired on the day of nAMD diagnosis (median difference: –1 µm; mean interval: 18.81 months). Moreover, no significant differences were found between the study eye and the fellow eye for the choroidal parameters analysed.

## Discussion

In this study, we investigated the relationship between choroidal structure and retinal fluid location in treatment-naïve nAMD patients with type 1 MNV. Choroidal parameters, including foveal choroidal thickness and LCVI, were significantly correlated with both SRF and IRF. Greater SRF was associated with increased foveal choroidal thickness and larger choroidal vessels in the foveal region, whereas IRF was associated with decreased foveal choroidal thickness and smaller choroidal vessels in the macular region.

Historically, retinal fluid in nAMD has been a primary biomarker for disease activity, guiding vascular endothelial growth factor (VEGF) inhibitor therapy with the goal of achieving complete resolution of both IRF and SRF [12]. Although traditionally considered equivalent in clinical management, emerging evidence indicates that IRF and SRF have distinct impacts on visual outcomes, potentially reflecting different origins and MNV phenotypes [13]. Several studies have demonstrated that IRF is consistently associated with poorer visual acuity and an increased risk of fibrosis and atrophy [4]. In contrast, SRF, particularly when present in small amounts, may be tolerated without significant visual impairment and may even confer a protective effect against RPE atrophy [14]. Interestingly, SRF in type 1 MNV generally requires more intensive anti-angiogenic treatment than other MNV types [10]. The poorer response to anti-VEGF therapy and the relatively favourable prognosis associated with SRF suggest that it may be influenced by factors independent of the MNV. Recent studies in other retinal conditions, including central serous chorioretinopathy [5], persistent fluid following retinal detachment surgery [15], cavernous sinus thrombosis [5], and carotid-cavernous fistulas [16], have identified structural choroidal characteristics as key contributors to SRF accumulation. Specifically, thickened choroids with large-calibre vessels, higher CVI, and intervortex venous anastomoses are indicative of altered choroidal circulation [5, 17]. These features may reflect venous overload, promoting fluid accumulation in the subretinal space and impairing its reabsorption by the RPE.

Different types of MNV have been associated with distinct patterns of fluid distribution and choroidal thickness. Type 1 MNV, characterised by the thickest choroid, is the most prevalent, accounting for approximately two-thirds of nAMD cases [10, 18]. It is typically associated with SRF, present in 85.7% of our cohort, and often requires more intensive anti-VEGF treatment despite its relatively favourable long-term outcomes [10]. Therefore, in this study, we focused on type 1 MNV to investigate whether choroidal characteristics could explain the patterns of fluid distribution in nAMD patients. We found that patients with type 1 MNV and SRF had a significantly higher mean foveal choroidal thickness compared to patients with no SRF. It has been reported that choroidal thickness may increase in the presence of active MNV [19]. To determine whether our results were related to MNV, we analysed choroidal thickness before MNV development and found that the change in thickness was neither clinically nor statistically significant (median difference of −1 µm). Moreover, no significant differences in choroidal thickness were observed when compared with the fellow eye without MNV. These findings suggest that choroidal thickness may not primarily reflect a consequence of the neovascularisation process, but rather a pre-existing characteristic that may be associated with greater SRF accumulation. Notably, the comparable choroidal parameters between the affected eye and the unaffected fellow eye do not negate a contributory role for choroidal structure in SRF development. The formation of MNV remains the essential and initiating trigger for SRF, as it disrupts the outer blood-retinal barrier and allows leakage from immature, hyperpermeable neovessels. In this context, choroidal thickness may act as a modulating factor: in eyes with greater choroidal thickness and potentially higher interstitial hydrostatic pressure, the fluid leakage initiated by MNV is more readily transmitted into the subretinal space. In the absence of MNV, fellow eyes with comparable choroidal thickness do not develop SRF, which is consistent with a two-hit model in which both MNV formation and choroidal predisposition are required for clinically significant SRF accumulation.

To assess whether choroidal thickness influences the amount of SRF beyond its presence or absence, we studied its correlation with SRF area, excluding patients with no SRF, and found a positive correlation, with higher choroidal thickness associated with greater SRF area. Traditionally, the volume of fluid has been linked to MNV severity. However, our findings suggest that choroidal structure may also be associated with the extent of fluid accumulation, independently of MNV characteristics. This aligns with other researchers who have found, using deep learning, that MNV parameters play a minor role in the exudation process [20]. We also examined the correlation between choroidal thickness and maximum SRF height and subfoveal SRF height, finding a positive trend that was not statistically significant. This may be explained by the typical shallow spreading of the SRF, which makes it challenging to detect substantial differences, especially under the foveal area, where SRF is displaced by the MNV, as other authors have suggested [21].

Based on these findings, we hypothesise that thickened choroids may represent a congestive circulatory state in which increased hydrostatic pressure favours fluid leakage into the subretinal space initially induced by the MNV. Parameters such as the percentage of choroidal luminal area and the percentage of large choroidal vessel area, relative to macular choroidal area, can indicate the extent of choroidal venous overload. Accordingly, we found that foveal LCVI was positively correlated with maximum SRF height and SRF area, whereas no correlation was found with CVI. These results suggest that, for SRF accumulation in these patients, the presence of large dilated choroidal vessels may be more relevant than the total amount of choroidal vessels, which may also be present in healthy, highly vascularised choroids. Notably, these significant findings were observed only when LCVI was measured in the foveal area and not in the macular choroidal area, indicating that the size of vessels underlying the MNV, responsible for draining the neovessels, may be most relevant.

In contrast, IRF exhibited the opposite behaviour: patients with type 1 MNV and IRF had a significantly thinner mean foveal choroidal thickness compared to those without IRF. Similarly, IRF volume showed an inverse correlation with choroidal thickness and macular LCVI. We propose that IRF in nAMD patients, similar to other retinal pathologies such as diabetic macular oedema, retinal vein occlusion, and posterior uveitis, which predominantly feature IRF, may share an ischemic and inflammatory origin [22–25]. We hypothesise that eyes presenting with IRF are characterised by a more chronic, pro-ischemic, and pro-inflammatory environment. A thinner choroid may reflect a depletion of the vascular component, potentially associated with choroidal insufficiency and outer retinal hypoxia. Literature suggests that chronic hypoxia and ischemia can drive the upregulation of inflammatory cytokines (beyond VEGF alone) and impair the fluid-pumping function of Müller glial cells [26]. When Müller cell metabolism is hypothesised to be compromised by ischemia, their ability to maintain retinal dryness may be impaired, potentially resulting in IRF accumulation (degenerative/cystoid oedema) rather than purely hydrodynamic subretinal accumulation. In addition, with decreased choroidal arterial inflow, there is less blood volume reaching the choroidal bed to be drained, potentially limiting venous congestion; consequently, choroidal vessels may remain undilated or atrophic, which could account for the observed low LCVI and choroidal thinning in these eyes. Unlike SRF, which may be more influenced by choroidal venous overload. Furthermore, while the foveal location of larger choroidal vessels is more significant for SRF, given their direct influence on MNV flow and proximity to the disrupted blood-retinal barrier, for IRF, the presence of larger choroidal vessels in the foveal region may be less critical. Instead, the general state of the choroid, particularly a diffusely atrophic and ischemic choroid, likely serves as a more relevant indicator. This explains the inverse correlation with LCVI observed only in the macular choroidal area and not in the foveal region.

Our findings may explain why the majority of type 1 MNV non-responders have persistent SRF [10]. According to our hypothesis, patients with thickened choroids may exhibit increased fluid leakage from the choriocapillaris, potentially associated with elevated interstitial pressure even before MNV development. The disruption of the blood-retinal barrier and RPE damage associated with neovascularisation may then facilitate fluid accumulation under the retina. Thus, this fluid may have dual origins, the choroidal interstitial space and the MNV itself, whereas IRF may be associated primarily with MNV leakage and disruption of retinal fluid homeostasis in the context of hypoxia and inflammation. In this scenario, anti-VEGF treatment does not directly target the congestive choroidal circulation, which may account for cases of residual SRF. Nevertheless, these hypotheses should be interpreted cautiously, as the retrospective and observational design of the present study does not allow conclusions regarding treatment response or causality. To further explore this, we are conducting a longitudinal study to investigate whether non-responders exhibit characteristics of choroidal venous overload.

We acknowledge several limitations. The retrospective cross-sectional nature of this study prevents conclusions regarding longitudinal outcomes, such as the utility of choroidal structure as a biomarker for treatment response or visual prognosis. The relatively small sample size may have limited our power to detect statistically significant results. The absence of an age-matched healthy control cohort is a further limitation, as it precludes direct comparison of the observed choroidal thickness, CVI and LCVI values with normative data from individuals without macular pathology. Therefore, the observed associations should be interpreted as relative differences within the type 1 MNV cohort rather than as evidence of absolute abnormalities in choroidal structure. Additionally, some fluid quantification parameters were measured manually and are thus subject to operator skill variations. Data on symptom duration prior to diagnosis and axial length were not available in our dataset, which precluded their inclusion as covariates in the multivariate analyses and limited the ability to fully exclude disease chronicity as a confounding factor. Furthermore, strict image selection criteria designed to ensure choroidal-scleral interface visibility necessitated the exclusion of phenotypes with massive retinal thickening, accounting for the lower mean retinal thickness values observed in our cohort.

In conclusion, we have demonstrated that SRF and IRF are correlated with choroidal thickness, but in opposing directions, in nAMD patients with type 1 MNV. SRF was associated with thicker choroids and larger foveal choroidal vessels, while IRF was related to thinner choroids and a decreased density of large choroidal vessels. These findings suggest that fluid location is associated with choroidal structure and may reflect distinct underlying pathophysiological mechanisms. Whether these structural characteristics influence anti-VEGF treatment response remains to be determined in prospective longitudinal studies. Therefore, further studies are required to elucidate the utility of choroidal structure as a biomarker for treatment response and prognosis in nAMD patients.

## Summay

### What is known about this topic

- Previous studies have established that in neovascular age-related macular degeneration (nAMD), the presence of intraretinal fluid (IRF) and subretinal fluid (SRF) is a key biomarker of disease activity and treatment response. Although both have traditionally been treated as equivalent indicators of exudation, accumulating evidence shows that they have distinct prognostic implications: IRF is associated with poorer visual outcomes, fibrosis, and atrophy, whereas SRF is often compatible with stable or even better-preserved vision. However, the mechanisms underlying these differences were not fully understood. Some recent findings in other retinal diseases suggested that choroidal structural factors—such as venous congestion or vascular remodelling—might contribute to SRF accumulation, but their role in nAMD, particularly in type 1 choroidal neovascular membranes (CNVM), had not been clarified.

### What this study adds

- This study demonstrates that in treatment-naïve type 1 nAMD, SRF and IRF are linked to opposite choroidal structural profiles. SRF correlates positively with choroidal thickness and with the foveal large choroidal vessel index (LCVI), suggesting that choroidal venous overload and the presence of enlarged choroidal vessels may promote fluid accumulation under the retina. In contrast, IRF is associated with thinner choroids and reduced LCVI, consistent with choroidal atrophy and ischemia. These findings provide evidence that fluid location in nAMD reflects distinct underlying pathophysiological mechanisms rather than a single process of exudation. Understanding these differences could explain the variable response to anti-VEGF therapy and support a more personalised approach to managing nAMD based on choroidal characteristics.

## Data Availability

The datasets generated and analysed during the current study are not publicly available due to participant confidentiality and institutional data protection policies, but are available from the corresponding author upon reasonable request.
